# Camp Dysentery

**Published:** 1863-11

**Authors:** Jehu Little

**Affiliations:** Ass’t. Surgeon 24th Mo. Vols.


### CAMP DYSENTERY.

By JEHU LITTLE, Ass't. Surgeon 24th Mo. Vols.

For the last two months I have eagerly searched all the medical journals of recent publication that came within my reach, hoping to find something new in the pathology and treatment of dysentery, but I am sorry to be compelled to say that not a single report or article from any quarter concerning this interesting theme has met my attention. Perhaps this is because productions emanating from the pens of army surgeons seldom find their way into the columns of the journals, or the disease does not prevail to any extent in civil practice during the present year. But few complaints are more common and inveterate in military practice than this; east and west, north and south, summer and winter, in hospitals and in the field, it exists in some form or other, to harass and perplex; and surgeons in changing from one field of labor to another are sure of this, that whatever other disorders they may have to combat, dysentery will come in for a large share of their time and consideration. I do not entertain a doubt, from what I have seen and heard in more than two years' service, but that one-third the deaths and discharges that are continually thinning the ranks of our army are directly occasioned by this one disease. Everything the books say upon this topic has been read and re-read, all the plans of treatment described carefully resorted to, and yet there is a general dissatisfaction with results and an earnest and perpetual seeking for something new and a hoping that a surer mode of managing this terrible enemy to our brave soldiers may be discovered and made known at an early day.

Our brigade of twelve hundred men has been stationed at Union City, Tennessee, since the first of last August, and during this period about two hundred men have been treated for dysentery, an equal number for diarrhœa, forty for intermittent fever, mainly of the tertian type, eight for remittent fever, two for pneumonia, twenty-five for conjunctivitis and two for typhoid fever. This is a flat country with a good many marshes, an alluvial rich soil upon a heavy clay subsoil, extensive dense forests of splendid timber of every species and variety growing in the Mississippi valley, with an abundant and luxuriant vegetation of all kinds. The water is poor and scarce, obtained chiefly from wells twelve to thirty feet deep, with wooden curbs, when walled at all. There seems to be very little mineral of any kind in this section of country. Last winter and spring months and first summer month were very wet, rain falling constantly, so that farmers and planters put in smaller crops than common, leaving a deal of land to the undisputed and undisturbed possession of rank weeds and grass; but since the first of July there has been but little rain; warm, clear, sunny days and cool, foggy nights. Fruits of all sorts that grow in our country thrive very well here, and the yield this season is quite large.

The food of the men for the most part has been the ordinary army ration issued to our soldiers everywhere, except the full allowance of fresh vegetables and beef, and vinegar has not been furnished at this post.

The every day bill of fare is strong hot coffee, salt fried bacon, heavy, hot biscuits, and coarse brown sugar. During the greater portion of time the men have had accesss to lager beer and whiskey of inferior qualities, but there has not been a great deal of immoderate drinking, though the habit of taking a small quantity every day has been pretty general; and I am not now prepared to assert that those who drank most suffered greatest from the malady in question.

The duties have been of the most active and exciting, the men being on guard, picket or scouting duty twenty-four hours in the forty-eight, and in either case exposed at night with no shelter but the thick foliage of the trees, and no bed save one or two blankets, and in the meantime drilling and doing camp duty. The men comprising this brigade are old soldiers, most of them having been in the service since the autumn of 1861, have served in the States of Missouri, Arkansas, Mississippi and Tennessee, and are thoroughly acclimated and seasoned to camp life. I believe I have now mentioned every circumstance necessary to give a person a tolerably correct idea of most agents exercising modifying influences, and to enable him to judge for himself what the remote and exciting causes of the disorder are in our case.

*Symptoms.*—Thsre is nothing new or peculiar in the symptoms worthy of naming; hot, harsh skin; dry tongue, with a moderately heavy yellowish coat and reddish about the edges; pulse a little excited and strong; urine scanty and of a pale yellow or red color; aching in the region of the kidneys; nausea and sickness at the stomach, and occasionally vomiting the contents mixed with bile; griping and pain in the umbilicus and hypogastrium; urgent calls to stool with excessive enesmus, and nothing passing from the anus but a spoonful or two of mucus and slime streaked with blood, sometimes half blood, of the consistence of jelly; dizziness and swimming of the head, and general lassitude and feebleness are the ever present signs of this disease in its fully developed stages. A manifest and general deficiency of glandular secretion is invariably present.

*Treatment.*—I believe all authors and practitioners recognize two distinct and opposite systems of treatment in the simple acute variety of this complaint, the astringent and the eliminative; but in managing the complicated varieties it becomes essential to take a wider therapeutical range and often employ agents belonging to both, and to neither of these systems. In civil practice, I almost always practiced the astringent system with good success, administering any of the most valuable and powerful of the vegetable or mineral astringents best suited to the case, and on entering the army very naturally began on this method, but with poor success, and after an impartial trial abandoned it and adopted the eliminative plan. It is very true, and worthy of remark, that the class of patients is different in civil practice to that in the army; in one case mostly children and women, and in the other all men; and then the surroundings are widely opposite.

As the disease does not come under our observation here till the third or fourth day, when it is completely established, the men never reporting themselves sick until too ill for duty, I commence at once with the most reliable remedial course of management, prescribing one of the following formulæ, the one considered best adapted to the particular case:

1. ℞. Ol. Ricini, ℥i; Ol. Turpentine, ʒi; Tinct. Opii, ʒiss; Fld. Ext. Ginger, ʒ⅔. M.

2. ℞. Sulph. Mag., ℥i; Aquæ, f ℥iv; Arom. Sulph. Acid, ʒi; Tinct. Opii, ʒij; Tinct. Peppermint, ʒ⅔. M.

3. ℞. Pulv. Rhei, ℈iss; Pulv. Ipecac; Pulv. Opii; Sub. Mur. Hyd. *aa* gr. v; Comp. Ext. Colocynth, gr. vi. M. ft. pil No. X.

Shake the medicine well and give a teaspoonful of the first every two, three or four hours till the desired effect is produced, namely, free and easy operations from the bowels with no pain, tenesmus or blood, and until the alimentary canal is well purged, when a general amendment of all the symptoms will be apparent. A tablespoonful of the second is given every three or four hours and continued till all the more violent symptoms abate and a like improvement supervenes.

One of the pills of the third formula is to be taken every three or four hours till the evacuations are more copious and easy and of a billious character without any blood, or slimy appearances. I often prescribe the common compound cathartic pills, one to be taken every four hours until the wished for result is witnessed.

In the meanwhile the patient is well washed or bathed with warm water, containing potash or soda, as often as deemed proper; kept warm and comfortable, and allowed but a little bland, nutritious, and easily digested food. And I would remark in this connection, that the subject of regimen cannot receive too close attention in affections of the alimentary organs. Warm cloths, sinapisms or fomentations of hops or peach tree leaves, or bitter herbs of some sort are placed upon the bowels over the seat of pain, when there is much distress. Absolute rest in a horizontal position is strictly enjoined when the disease is at its height. When the patient is seen early, a solution of Chlorate of Potassa and Sulphate of Morphia, say one drachm of the former, one grain of the latter, and four ounces of water, a tablespoonful to be taken every three or four hours, will seldom fail to perform a cure if a few simple hygienic rules are observed.

When there is laxity of the bowels after the disorder is cured, I direct one of these pills three times a day: ℞—Sulph. Iron, Sulph. Quinia, Pulv. Ginger, *aa* gr. v; Ext. Gentian, gr. x. M. ft. pil. No. v. Mucilaginous drinks are freely administered during the whole course of treatment if there is much thirst, and they are excellent from time to time when there is no demand for fluids. I would by no means discard injections in treating this complaint, because in some cases their value is inestimable and they should not be dispensed with, as their timely and cautious administration prevents much suffering. When there is much irritation and uneasiness in the rectum and lower portion of the abdomen, and almost continued tenesmus and incessant demands to go to stool, accompanied with prostration, an injection composed of eight grains of Acetate of Lead, two grains of Sulphate of Morphia, three or four tablespoonsful of good starch and two or three ounces of warm water acts admirably and accomplishes all that could be desired. The Nitro-Muratic bath and the Muriate of Ammonia, in certain cases, are efficient remedies, and their good effects are truly gratifying. When convalescence is fully established, Quinine, Iron, Brandy, Wine and good living soon restore the patient to his former health and strength; generally from fifteen to twenty days from the time he was taken under treatment he is able for duty.

Before commencing the medical treatment of any disease a correct and thorough diagnosis should be formed, and during the progress of the malady every shadow of variation and change should be minutely noted from day to day and the management conducted accordingly. The indications of treatment in Dysentery are clearly three-fold to arouse glandular secretions and expel them from the system, to quiet undue and morbid excitement, and to gently stimulate all the tissues of the organism, and the foregoing prescriptions are designed to fulfill all these indications. All violent and perturbative agents are studiously excluded and the strength of the sick is conserved as much as possible; the principles and practices of hygiene are continually kept in view and insisted upon.

I have hurriedly and roughly given an outline of my plan of treating Dysentery in the army, and I cordially and confidently recommend it to all who pursue a different mode without the satisfaction they wish, as it has proven remarkably efficacious in my hands.

